# Review of Mass Drug Administration for Malaria and Its Operational Challenges

**DOI:** 10.4269/ajtmh.14-0254

**Published:** 2015-07-08

**Authors:** Gretchen Newby, Jimee Hwang, Kadiatou Koita, Ingrid Chen, Brian Greenwood, Lorenz von Seidlein, G. Dennis Shanks, Laurence Slutsker, S. Patrick Kachur, Jennifer Wegbreit, Matthew M. Ippolito, Eugenie Poirot, Roly Gosling

**Affiliations:** The Malaria Elimination Initiative, Global Health Group, University of California, San Francisco, California; Malaria Branch and Division of Parasitic Diseases and Malaria, Centers for Disease Control and Prevention, Atlanta, Georgia; London School of Hygiene and Tropical Medicine, London, United Kingdom; Mahidol-Oxford Tropical Medicine Research Unit, Bangkok, Thailand; Australian Army Malaria Institute, Enoggera, Australia; Division of Infectious Diseases, Johns Hopkins University School of Medicine, Baltimore, Maryland

## Abstract

Mass drug administration (MDA) was a component of many malaria programs during the eradication era, but later was seldomly deployed due to concerns regarding efficacy and feasibility and fear of accelerating drug resistance. Recently, however, there has been renewed interest in the role of MDA as an elimination tool. Following a 2013 Cochrane Review that focused on the quantitative effects of malaria MDA, we have conducted a systematic, qualitative review of published, unpublished, and gray literature documenting past MDA experiences. We have also consulted with field experts, using their historical experience to provide an informed, contextual perspective on the role of MDA in malaria elimination. Substantial knowledge gaps remain and more research is necessary, particularly on optimal target population size, methods to improve coverage, and primaquine safety. Despite these gaps, MDA has been used successfully to control and eliminate *Plasmodium falciparum* and *P. vivax* malaria in the past, and should be considered as part of a comprehensive malaria elimination strategy in specific settings.

## Introduction

Mass drug administration (MDA) was a component of many malaria elimination programs during the mid-twentieth century eradication era, but since then the malaria community has viewed it with skepticism due to concerns regarding its efficacy, sustainability, and operational feasibility, a lack of clear objectives for MDA programs, and fear of accelerating drug resistance.^1^ However, in light of the availability of antimalarials with transmission-reducing effects (e.g., artemisinin-based combination therapies [ACTs] and primaquine) and the limitations of current diagnostic tools to detect sub-patent infections, the role of MDA as an elimination tool needs to be reexamined.^2^,^3^ Many field studies and programmatic implementations of MDA have been carried out over the past century with varying degrees of success. An initial review of this heterogeneous body of work was conducted in 2003,^4^ and a Cochrane Review focusing on the quantitative effects of malaria MDA was published in December 2013.^5^ To build on these reviews and maximize understanding of the key factors for success in previous MDA campaigns, we have conducted a qualitative analysis of published, unpublished, and gray literature, supplemented with consultations with malaria MDA experts. A summary of our primary findings and the remaining knowledge gaps are presented here, to serve as the basis for improved design and implementation of MDA in malaria elimination and eradication programs.

## Methods

We conducted a comprehensive literature review and held consultations with experts to thoroughly document current and past experiences with MDA. The literature search originated from the Cochrane Review,^5^ in which 3,048 studies were identified for screening. We assessed 240 of these studies, applying several exclusion criteria (see Appendices A and B). We included a wide range of chemoprevention studies of varying quality with the aim of obtaining essential information on operational details and other study features that were excluded from the Cochrane Review. Thus, studies that treated subgroups rather than entire populations as well as those that used subtherapeutic drug doses were included. Data from all included studies were systematically extracted and entered into a Qualtrics (Qualtrics, Provo, UT) database for analysis.^6^

We identified experts with experience in conducting MDA for malaria and other vector-borne diseases through published literature and recommendations from colleagues. Input was solicited from any experts interested in participating, regardless of when or where their MDA experience occurred, the outcomes of their fieldwork, or opinions on MDA. Efforts were made to interview experts with experience in a wide range of geographical and endemicity settings. Individual consultations were conducted in person, by telephone, or by e-mail using a semi-structured questionnaire that covered primary MDA topic areas to guide the discussion, and included requests for any unpublished reports and gray literature that experts were willing to share.

## Results

After applying the exclusion criteria described in the appendices and dividing some studies into sub-studies to facilitate data extraction, we analyzed a total of 182 published accounts of MDA. These studies were carried out all over the world and span the whole of the past century, with the earliest published in 1913 and the most recent in 2011. Several reports were obtained through expert consultations, documenting the implementation of MDA campaigns in the field that had not been published in English language academic journals. These unpublished reports included campaigns that took place in Nissan, Papua New Guinea in the 1960s,^7^ the Solomon Islands in the late 1980s, and Comoros in the mid-2000s. Additional reports documenting work in Afghanistan, Azerbaijan, Democratic People's Republic of Korea (DPRK), Tajikistan, and China have since been published.^8^,^9^ In addition to this documented work, the 15 MDA experts shared personal experiences with implementing MDA for malaria and other vector-borne diseases in Africa (Burkina Faso, Ethiopia, The Gambia, Liberia, Nigeria, and Senegal), Asia Pacific (Greater Mekong subregion and Vanuatu), and the Americas (Ecuador and Guatemala). Nonresponses to consultation requests led to under-representation from the Americas and eastern Mediterranean regions.

The data extracted from the literature review, unpublished/gray literature, and qualitative consultations were analyzed by topic, including operational details, contextual characteristics, and drug regimen.^10^ As established in the Cochrane Review, the studies varied considerably in terms of design, rigor, and depth and quality of data, limiting their analysis and comparability. Qualitative data obtained through expert consultations were therefore essential to gain a more comprehensive understanding of past MDA experiences and provide important contextual background for the findings of the review.

### Characteristics of successful studies.

Many of the published studies were deemed successful by the investigators, although most did not define their objectives clearly, making it difficult to assess whether study goals were actually achieved. Despite this lack of clarity, we identified 12 studies that met a definition of success applicable to malaria elimination settings: zero indigenous malaria cases in the target population maintained for at least 6 months after the end of all drug administration (Table 1). The majority of the published studies (63%) had a follow-up period of less than 6 months, preventing an assessment of the interventions' long-term effects on transmission. Many studies were able to reduce parasite prevalence in the target population temporarily, but either were not able to reach zero prevalence or were followed by an increase in prevalence shortly after drug administration ceased, a finding echoed in the Cochrane Review.^5^

The primary factors determining the success of MDA mentioned almost universally by MDA experts were: achieving at least 80% or even 90% coverage of the target population with drug administration, directly observed treatment (DOT), strong community engagement, high coverage with concomitant vector control interventions, and the use of 8-aminoquinolines, particularly in *Plasmodium vivax* transmission settings.

### Delivery methods and community engagement.

The published studies did not thoroughly or consistently explain their delivery and community engagement strategies, but those that did used a wide range of approaches. In the studies that involved DOT (58%), drug distribution and observation was performed by community volunteers, local health workers, study authors, and/or external organizations. DOT was not limited to small-scale MDA; while most studies targeted populations in the hundreds or thousands, several campaigns implemented DOT among populations in the hundreds of thousands and even millions. Table 2 summarizes the MDA campaigns that were conducted on a large scale, covering 100,000 people or more. In addition, DOT has been implemented not just for single-dose treatment but also for complicated, multiday drug regimens. For example, in Palestine, a population of 1,257 villagers was treated twice daily for 5 days over three monthly rounds.^11^ Through DOT, maintenance of treatment lists, and careful follow-up, study authors were able to treat 81% of the population with all three rounds of MDA. The number of rounds of drug administration varied widely among the successful studies, from just one to numerous rounds distributed over 2 months to 10 years.

Other strategies leading to effective MDA included population censuses and the mobilization of local workers to monitor the movement of people in and out of a study area. One study described the use of incentives for community participation and adherence to the full MDA regimen, specifically lottery tickets for prizes (sewing machines, bicycles, etc.).^12^ Six of the 12 successful studies used DOT with trained volunteers or the study authors themselves, whereas delivery methods in the other studies were not described. Different types of community engagement were used in the successful studies, namely, working with community leaders and elders to ensure cooperation, extensive health education and outreach, and active participation through the formation of volunteer malaria teams. Two study authors specifically noted that strong community participation was crucial for success.^13^,^14^

MDA experts stated that delivery methods involving house-to-house visits are preferred over distribution at centralized, fixed-point locations when logistically feasible, to ensure high coverage. Local health workers or volunteers should be used for drug distribution, since they understand the environment and local customs, and can garner more trust and acceptance among their peers than outsiders. In areas with low transmission, experts recommended working with older members of the community who may remember when malaria was more prevalent and be more committed to preventing its return.

The unpublished account of MDA in the Solomon Islands is a prime example of the importance of securing community buy-in to achieve high coverage. This project targeted a population of around 30,000 in the capital city and was thoroughly planned, well staffed with local workers, and involved mass media community outreach to encourage participation, yet coverage was still only 67%. Investigators believed that this was due to refusal of the targeted population to take multiple rounds of drugs when they were not ill. In comparison, the MDA carried out in Nissan, Papua New Guinea was notable for achieving nearly 100% coverage, attributed by the investigators to a high degree of community cooperation and a strong health infrastructure that facilitated intense screening of all arrivals to the island.^7^

### Co-interventions.

Co-interventions were deployed in nearly half of the studies. Of these, 65% conducted indoor residual spraying (IRS), primarily using dichlorodiphenyltrichloroethane (DDT), 33% conducted chemical or biological larval control, 23% carried out environmental management (e.g., vegetation clearing, waterway construction), and 16% distributed bed nets, treated or untreated. Ten of the 12 successful studies implemented vector control co-interventions: eight used IRS, three used insecticide-treated nets, and two used multiple measures. Experts agreed that vector control is essential and should be used prior to commencement of, or concurrently with, MDA to bring transmission down to low levels.

### 8-aminoquinoline-based drug regimens.

Drug regimens used in the published studies were diverse, and varied depending on location and timeframe as well as on biological concerns, including those related to the prevalence of glucose-6-phosphate dehydrogenase (G6PD) deficiency. 8-aminoquinolines were included in 69 of the studies (38%), five of which were monotherapies with either plasmoquine or primaquine. Six of the 12 successful studies included 8-aminoquinolines in combination with other drugs. All but one of the unpublished reports included primaquine, distributed as monotherapy or in combination with artemisinin derivatives and other blood schizonticides. 8-aminoquinolines were used for both *P. falciparum* and *P. vivax* MDA programs, although exact regimens varied according to targeted species. A selection of primaquine-containing dosing regimens is shown in Table 3.

Only eight studies that included 8-aminoquinolines documented the prevalence of G6PD deficiency in the target population (ranging from 1% to 39%), and just three of those studies documented drug safety protocols. For example, in China and DPRK, patients with a history of hemolysis were excluded from future treatments, whereas in Afghanistan and Azerbaijan, a modified drug regimen was implemented in which the 14-day course of primaquine treatment was interrupted and drugs were not given on days 5–7. Because this intermittent schedule was thought to disrupt the hemolytic effects of the drug, it was deemed safe for populations with a high prevalence of G6PD deficiency.^8^ Regardless of drug and dosing regimen, no MDA-related deaths were documented during these primaquine-based campaigns; patients who experienced adverse events recovered with routine supportive care, and no long-term hospitalization or blood transfusions were necessary, according to investigators' records. However, it should be noted that none of these studies reported using an active pharmacovigilance system.

Few studies included details on subpopulations excluded from MDA for safety reasons. When they were described, the most common excluded groups were infants and young children. Other excluded groups, described infrequently, included pregnant women, subjects recently treated for malaria, and people with chronic illness. Fewer than 10% of the studies that included 8-aminoquinolines explicitly noted the exclusion of pregnant women; none of the studies reviewed reported on pregnancy outcomes when discussing adverse events.

MDA experts universally believed that inclusion of an 8-aminoquinoline in the drug regimen, either primaquine or tafenoquine, was essential for eliminating the last reservoirs of infection. They noted that patient monitoring is critical for quickly identifying subjects with hemolysis, and medical interventions, including blood transfusion, should be readily accessible.

### *P. vivax* elimination.

A primary finding of the Cochrane Review was that MDA had a greater impact on *P. falciparum* transmission than on that of *P. vivax* in a given study using the same drug regimens, which did not always include an 8-aminoquinoline. Our qualitative analysis of a much broader range of MDA campaigns than that covered by the Cochrane Review reveals strong evidence that MDA with an 8-aminoquinoline is an effective intervention against *P. vivax*, particularly as an outbreak response in highly seasonal transmission settings. The study carried out in Jiangsu Province, China in the 1970s describes the use of primaquine to reduce *P. vivax* transmission on a massive scale.^9^ Entire counties, nearly 30 million people in total, were given directly observed, seasonal “spring treatment” by teams of community health workers and local public health officers prior to the onset of the transmission season, largely in the absence of vector control measures. According to the few records available, the incidence of severe adverse events was negligible and no deaths were reported, despite the enormous scope of primaquine distribution. After 10 years of MDA, the annual parasite index of *P. vivax* in Jiangsu Province dropped from 113.6 to 2.1 per 1,000 population.

Other examples of successful control of *P. vivax* epidemics with primaquine-based MDA are found in the series of recently published accounts from Afghanistan, Azerbaijan, DPRK, and Tajikistan.^8^ In these countries, an approach called mass primaquine prophylactic treatment (MPPT) was used, consisting of closely monitored 14-day courses of primaquine as monotherapy on an annual basis, ranging from three to 13 rounds. As in Jiangsu Province, reports of adverse events were rare: across all locations and years, less than 4% of nearly 9 million people treated experienced adverse events, with no blood transfusions reported. Unlike the Jiangsu account, vector control interventions were emphasized as important for the success of MPPT. However, due to a lack of resources, vector control activities in some areas were often of poor quality and achieved only minimal coverage, according to the investigators. Despite these problems, considerable case reductions and halting of *P. vivax* epidemics were seen in Afghanistan, Azerbaijan, and DPRK, all of which were able to achieve drug coverage of over 90% in populations ranging from 24,000 to 500,000. In Tajikistan, the effects of MPPT were not as pronounced, and this was attributed by the study authors to the fact that population coverage never exceeded 80%.

## Discussion

This extensive review of published, unpublished, and gray literature has revealed a wealth of information supporting the use of MDA as an intervention for malaria elimination. Selected points summarized here address key factors for successful design and implementation of MDA, and highlight important gaps in knowledge that need to be addressed to move toward broader implementation of MDA as part of malaria elimination programs.

Our review has shown that in the majority of previous studies, MDA reduced parasite prevalence (or other measures of transmission) only temporarily, and transmission returned to pre-intervention levels shortly after drug administration concluded. In these settings, this occurred even after multiple rounds of MDA were combined with vector control or included an 8-aminoquinoline. However, we also identified several examples in which MDA interrupted malaria transmission. In general, we found that implementing MDA in higher endemicity settings will reduce transmission, but there is a much better chance of interrupting transmission when MDA is implemented in areas of low endemicity in combination with other interventions, findings that were echoed in the Cochrane Review. Malaria was successfully eliminated from Aneityum,^14^ Lanyu,^15^ and Nissan,^7^ small island settings where ports of entry were controlled and population movement was closely monitored. Similar success was seen in village settings that were relatively remote, geographically.^13^,^16^,^17^ Yet achieving elimination was not limited to small, isolated populations. Transmission was sometimes interrupted even when much larger groups were targeted with MDA,^18^–^21^ indicating that delivery strategies and intervention combinations may be more critical determinants of success than population size.

An additional factor favoring success is an ability to adapt an MDA strategy to changing circumstances. This was demonstrated in Cambodia, where it was observed that parasite rates persisted in some villages after receiving MDA.^13^ In response, investigators revised their study protocol, adding a second round of treatment in those villages, and replacing local malaria workers to improve drug distribution processes. These changes resulted in a significant drop in parasite rates. A flexible approach to MDA that takes into account the unique, and constantly evolving, local patterns of human movement, transmission dynamics, and vector populations is essential.

All consulted experts saw DOT as a vital part of a delivery strategy, and we found evidence that DOT using multiday drug regimens is possible on a large scale. In the successful campaigns, large populations were treated as small units. For example, MPPT delivery teams were assigned groups of approximately 200–250 people within a larger campaign targeting populations in the tens of thousands to millions.^8^ Targeting small subgroups allowed for more efficient use of limited resources, and facilitated safety monitoring and achievement of coverage higher than 80%, a minimum cutoff recommended by the consulted experts and used in recent mathematical modeling research addressing malaria elimination.^22^,^23^

Another significant finding was that community participation, understanding, and acceptance are essential for success. In the countries where MPPT was implemented,^8^ obtaining support of local authorities and cooperation of communities was noted as key for ensuring good coverage and efficiency of drug distribution. In the Solomon Islands, where great efforts were made to educate and engage the community using mass communication, investigators believed that MDA failed because people were unwilling to take drugs on repeated occasions when they were not sick. A more intense MDA program in nearby Vanuatu, however, succeeded in interrupting transmission when the community was engaged on a more direct, interpersonal level.^14^ A clear understanding of local culture, values, and community structure will improve outreach and education efforts, thus increasing participation.

Despite inconsistent use of vector and larval control in the reviewed studies, the vast majority of successful campaigns used co-interventions to bring malaria incidence down to a low level before attempting to interrupt transmission with MDA, or both interventions were initiated simultaneously. Although two of the 12 successful studies were able to achieve zero transmission in some areas without implementing co-interventions, one study author acknowledged that without vector control, the population remained vulnerable to future outbreaks arising from a residual parasite pool in asymptomatic patients, or due to importation from neighboring villages.^13^ The other study contained very limited details, so contributing factors for the success of MDA in the absence of vector control cannot be adequately assessed.^18^ Vector control is less of a priority in highly seasonal *P. vivax* settings where there are regular periods of zero transmission with no vectors present, but experts agreed that in other transmission settings, every effort should be made to minimize vector–human contact either prior to or concurrent with MDA implementation. Vector control should be included as a central part of an MDA strategy, particularly for *P. falciparum* elimination in higher transmission areas.

Although millions of doses of primaquine have been distributed on a massive scale in several countries with minimal adverse events reported, more safety data on both primaquine and tafenoquine regimens are needed to support their wider implementation as part of an elimination strategy.^24^,^25^ Past studies did not document rigorous pharmacovigilance activities, and it is very likely that adverse events were underreported. Studies on the safety of primaquine in G6PD-replete and G6PD-deficient populations are currently underway. Reassuringly, a recently completed review of evidence derived from previous studies involving 8-aminoquinolines by the WHO^26^ concluded that there is a very low risk of hemolysis among subjects with mild or moderate G6PD deficiency when given a single, low dose of primaquine. Safety studies among pregnant women are also needed, given the dearth of data on pregnancy outcomes following primaquine administration. A side study related to MDA conducted in the Gambia^27^,^28^ examined the safety of artesunate and pyrimethamine-sulfadoxine in pregnancy and found no harmful effects; more studies using the methodology applied in the Gambia are necessary.

In addition, more data on the most appropriate MDA drug regimen are needed. The benefits of adding single, low-dose primaquine for *P. falciparum* or single-dose tafenoquine to ACTs for both *P. falciparum* and *P. vivax* need to be explored through drug efficacy trials, as should the addition of other potential anti-transmission agents, such as ivermectin and methylene blue, and new drugs as they become available.^3^

The lessons and knowledge gaps summarized in this review should be addressed through the establishment of a global research agenda, and in light of the current funding crisis for health research, a pragmatic and efficient approach must be taken. Differences in the efficacy of various drug regimens are likely to have less of an impact on outcome than ensuring high coverage^29^ during an MDA campaign; therefore, large-scale trials should focus on optimal delivery strategies for MDA, as well as the monitoring and evaluation of coverage, cost-effectiveness, and impact in different endemicity settings. Small-scale, highly controlled trials can be used to identify the best MDA drug regimens and potential vector control co-interventions for use in the larger trials. The target objectives of the recommended MDA trials must be well defined prior to implementation to bring much-needed clarity and focus to this area of research.

### Limitations.

Although the research conducted for this review was comprehensive, significant limitations exist. The high variability of study methods and settings as well as the poor quality of data derived from the published literature pose major difficulties for analysis and the drawing of firm, generalizable conclusions based on study results. Combining the many and somewhat disparate forms of MDA into one review may have introduced additional heterogeneity and weakened the overall conclusions on effect and success of MDA interventions. However, the more selective Cochrane Review supports the findings of this review, and by including a wider variety of studies along with input from MDA experts, a broader assessment of factors such as delivery strategies and size of target population could be carried out. These experts provided valuable contextual insights based on decades of institutional knowledge and personal experience with MDA. Yet, their input cannot be treated in the same manner as data derived from a rigorous published study, and the experts we consulted tended to be biased in support of MDA. In addition, not everyone who has experience with MDA was interviewed, and the search for unpublished work was not exhaustive, introducing reporting bias to these findings. Publication bias was not formally assessed, but may have influenced the review findings because malaria programs are less likely to publish studies with negative outcomes and more likely to downplay adverse events. Finally, the definition of success used to evaluate the published studies is more applicable to *P. falciparum* than *P. vivax*. The 6-month transmission-free period is not sufficient for assessing clearance of hypnozoites, the dormant liver stage of *P. vivax.*

## Conclusion

On the basis of the evidence presented here, MDA for malaria should be designed and implemented with a long-term view and a contextual, adaptable approach, drawing on local knowledge and evidence as well as lessons learned from previous MDA experiences. The historical successes described in this review demonstrate that MDA can be used to reduce and, in some circumstances, interrupt transmission of both *P. falciparum* and *P. vivax* malaria in specific settings, and should be considered for operational implementation as one component of a comprehensive elimination strategy. In areas with low transmission or seasonal *P. vivax* transmission, any MDA strategies used should include close monitoring for coverage, safety, and population impact on transmission rates. In higher transmission areas, more research is likely needed before MDA can be widely adopted, focusing particularly on additional measures needed to maintain zero or low transmission following treatment rounds.

## Supplementary Material

Supplemental Appendix.

## Figures and Tables

**Table 1:** Studies that interrupted transmission for over 6 months after the end of MDA

Study author Country Years	Type and goal of MDA	Drug regimen and duration of intervention	Target population description	Method of delivery (no. of treatment days)	Target population size (coverage %)	Parasite species	Additional control measures	Outcomes at conclusion of intervention
MDA for malaria outbreak response								
Lakshmana-charyulu^21^ India1961	Mass treatment: outbreak response	Two rounds CQ + PYR (dosage ND)	All individuals	ND (1 day for two rounds)	30,000–35,000 (80)	Pf and Pv	IRS	MDA brought PP close to 0; IRS and surveillance over next 4 years led to elimination
		Total duration: 4 months						
Singh^30^ India 1962–1964	Mass treatment: outbreak response	PQ 15 mg daily for 5 days + CQ 600 mg single dose for four rounds; first round treated everyone, subsequent rounds targeted only febrile cases and their contacts	All individuals (except infants, pregnant, seriously ill)	DOT (5 days, for up to four rounds)	22,369 cumulative over 2 years (average 76)	Pf and Pv	IRS	MDA and IRS combined controlled outbreak and brought PP to 0
		Total duration: 2 years						
Liu^19^ China 1981–1985	Mass treatment: outbreak response	During low transmission season: CQ 1,200 mg + PQ 180 mg over 8 days During high transmission season: CQ 300 mg + PQ 30 mg twice a month	All individuals	ND (∼20 days per year for 5 years)	26,369 (100)	ND	Bed nets	Transmission-interrupted PP reduced to 0.05%
		Total duration: 5 years						
MDA for malaria morbidity reduction and elimination								
Dupoux^18^ Tunisia 1936	Mass chemoprophylaxis: reduce morbidity	Premaline (dosage ND) every 10 days for 1 month, then every 14 days for 5 months	All individuals	DOT (∼13 days)	27,097 (100)	ND	None	Transmission interrupted with parasite indices reduced to 0 in some areas
		Total duration: 6 months						
Berberian^16^ Lebanon 1946–1947	MSAT, then mass treatment: reduce morbidity	CQ 125–500 mg weekly	All individuals ≥ 6 months of age	DOT (1 day for 20–32 rounds)	93–215 (100)	Pf and Pv	IRS immediately after conclusion of MDA	Case incidence decreased to 0.02/1,000 persons/month; cases were suspected relapsed Pv infections
		Total duration: 8 months						
Department of Health Taiwan^15^ Taiwan 1955	Mass treatment: elimination	CQ 12 mg/kg single dose	All individuals (except infants)	DOT (1 day)	1,502 (ND)	Pf, Pv, and Pm	IRS	Neither MDA nor IRS alone able to bring PP to 0; combined interventions led to elimination
		Total duration: 2 months						
De Zulueta^31^ Uganda 1960	Mass treatment: elimination	CQ 200–600 mg + PYR 16.5–49.5 mg, two single doses ∼6 months apart	All individuals ≥ 3 months	ND (1 day for two rounds)	8,000–16,000 (50–100)	Pf and Pm	IRS	Transmission interrupted
		Total duration: 6 months						
Huehne^17^ Malaysia Orang Asli 1961–1963	Mass treatment: elimination	CQ 600 mg + 49.5 mg PYR monthly dose	All individuals	ND (1 day for 31 rounds)	147 (50–75)	Pf	IRS	IRS + MDA brought PP to 0; outbreak 13 months after MDA ended was due to imported case
		Total duration: 31 months						
Huehne^17^ Malaysia Coastal belt 1961–1963	Mass treatment: elimination	CQ 600 mg + 49.5 mg PYR approximately every 6 months	All individuals	ND (1 day for four rounds)	ND	Pf	IRS	MDA ceased after four rounds with no transmission; interruption of transmission attributed to IRS, with MDA hastening progress
		Total duration: 24 months						
Dapeng^20^ China 1985–1994	Mass treatment: elimination	CQ 1,500 mg + PQ 90 mg once annually for 3 consecutive days	All individuals where incidence was ≥ 5% in previous season	ND (3 days for 10 rounds)	1,052,170 cumulative over 10 years (ND)	Pf and Pv	IRS, bed nets	Pf incidence reduced to 0 and Pv incidence to 0.05/1,000 persons/month; success attributed to vector control interventions
		Total duration: 10 years						
Kaneko^14^ Vanuatu 1991	Mass treatment: elimination	CQ 600 mg + SP 1,500 mg/75 mg + PQ 45 mg weekly dose in weeks 1, 5, and 9; CQ 300 mg + PQ 45 mg weekly dose in weeks 2–4 and 6–8	All individuals (pregnant women CQ only; no PQ for infants < 3 months)	DOT (1 day for nine rounds)	718 (90)	Pf, Pv, and Pm	Bed nets, larviciding, community health education	Malaria eliminated from Aneityum
		Total duration: 9 weeks						
Song^13^ Cambodia 2003–2004	Mass treatment: elimination	Artemisinin-piperaquine 24–750 mg, two doses given at 0 and 24 hours (second round 1 year later in some villages) + PQ 9 mg every 10 days for 6 consecutive months	All individuals ≥ 1 year	DOT (19–20 days)	2,387–3,653 (ND)	Pf, Pv, and Pm	None	PP reduced to 0 in some villages; gametocytemia reduced to 0.6% after 1 year follow-up
		Total duration: 6–12 months						

CQ = chloroquine; DOT = directly observed treatment; IRS = indoor residual spraying; MDA = mass drug administration; MSAT = mass screen and treat; ND = not described; Pf = *Plasmodium falciparum*; Pm = *Plasmodium malariae*; PP = parasite prevalence; PQ = primaquine; Pv = *Plasmodium vivax*; PYR = pyrimethamine; SP = sulfadoxine-pyrimethamine.

**Table 2:** Large-scale MDAs targeting populations > 100,000

Study author Country Years	Type and goal of MDA	Drug regimen and duration of intervention	Target population description	Method of delivery (no. of treatment days)	Target population size (coverage %)	Parasite species	Additional control measures	Outcomes at conclusion of intervention
Joncour^32^ Madagascar 1949–1955	Mass chemoprophylaxis: control and elimination	CQ 300 mg/week	All individuals age 0–13	DOT (1 day for 104 rounds)	760,000 (100)	Pf, Pv, and Pm	IRS, larviciding	Chemoprophylaxis decreased morbidity and mortality; PP was 10–35% among treated population and higher among untreated
		Total duration: 2 years						
Gabaldon^12^ Venezuela 1957–1958	Mass treatment**:** elimination	PYR 50 mg weekly	All individuals ≥ 1 month	DOT (1 day for 24 rounds)	111,995 (ND)	Pv	IRS, community participation incentives	MDA interrupted transmission, brought PP to 0% but failed to cure all Pv infections; transmission resumed after relapses occurred
		Total duration: 24 weeks						
Ossi^33^ Iraq 1963	Mass chemoprophylaxis: outbreak control and elimination	CQ 450 mg + PYR 50 mg twice per month	All individuals	ND (1 day for six rounds)	250,000 (80)	Pf and Pv	IRS, active case detection	MDA decreased morbidity but unsuccessful at interrupting autumn transmission
		Total duration: 3 months						
Dola^34^ Zanzibar 1968	Mass treatment**:** elimination	CQ 300 mg + camoquin 300 mg + PQ 30 mg every 2 months	All individuals	ND (1 day, no. of rounds ND)	124,065 (84)	Pf	None	MDA was ineffective: incidence increased to 10.5/1,000 persons/month vs. pre-intervention level of 9.7/1,000 persons/month
		Total duration: ND						
Kondrashin^8^ Azerbaijan 1971–1975	MPPT: elimination	PQ 15 mg daily for 14 days	All individuals (except infants, pregnant, chronically ill)	DOT (14 days for five rounds)	10,587–106,555 (87–93)	Pv	None	MPPT controlled epidemic; PP reduced to 0.7% and maintained for several years with only residual active foci
		Total duration: 5 years						
Hsiang^9^ Jiangsu 1973–1983	Seasonal mass treatment: control of epidemic	1973–1976: PQ 30 mg daily for 4 days + PYR 50 mg daily for 2 days	1973–1976: All individuals in rural counties	DOT (1973–1976: 4 days for four rounds; 1977–1983: 8 days for seven rounds)	1973–1976: 13,389,482–27,974,966 (ND)	Pv and minimal Pf	Bed nets (very low coverage), intermittent chemoprophylaxis	MDA decreased parasite reservoir but did not interrupt transmission; API dropped from 113.6 in 1973 to 2.1 in 1983
		Total duration: 4 years	1977–1983: All index cases from previous year and their contacts		1977–1983: 4,446,687–16,534,356 (ND)			
		1977–1983: PQ 22.5 mg + PYR 12.5 mg daily for 8 days						
		Total duration: 7 years						
Garfield^35^ Nicaragua 1981–1982	Mass treatment: control and elimination	CQ 350–1,500 mg + PQ 10–45 mg over 3 days	All individuals ≥ 1 year	ND (3 days)	1,900,000 (80)	Pf and Pv	Larviciding, breeding site reduction, community health education	Incidence of Pf declined for 7 months and Pv declined for 4 months; both then returned to pre-intervention levels
		Total duration: 3 days						
Dapeng^20^ China 1985–1994	Mass treatment: elimination	CQ 1,500 mg + PQ 90 mg once annually for 3 consecutive days	All individuals where incidence was ≥ 5% in previous season	ND (3 days for 10 rounds)	1,052,170 cumulative over 10 years (ND)	Pf and Pv	IRS, bed nets	Pf incidence reduced to 0 and Pv incidence to 0.05/1,000 persons/month; success attributed to vector control interventions
		Total duration: 10 years						
Han^36^ Republic of Korea 1997–2005	Mass chemoprophylaxis: control	CQ 300 mg weekly for active duty soldiers; PQ 14 mg/day for 14 days for soldiers on retirement	Active and retired soldiers	ND (1 day per week of service; then 14 days)	985,282 cumulative over 9 years (ND)	Pv	IRS, bed nets	Mass chemoprophylaxis reduced incidence to 0.08/1,000 persons/month among soldiers
		Total duration: 9 years						
Aliev^37^ Tajikistan 1998–1999	Mass treatment: outbreak control and elimination	PQ (dosage and regimen ND)	All individuals	DOT (ND)	257,200–421,000 (ND)	ND	IRS, larviciding	MDA reduced incidence to 0.56/1,000 persons/month but failed to interrupt transmission
		Total duration: 2 years						
Hsiang^9^ Jiangsu 2000–2009	Focal mass treatment: control of epidemic	CQ 400 mg daily for 3 days +	Index cases of past 1–2 years and all contacts (except < 3 years, pregnant, seriously ill)	DOT (8 days for up to 10 rounds)	1,863,399–1,926,183 (60–98)	Pv	IRS in some areas, bed nets (very low coverage)	Targeted MDA decreased API to 0 in some areas, but transmission was not fully interrupted
		PQ 22.5 mg daily for 8 days						
		Total duration: 10 years						
Kondrashin^8^ DPRK 2002–2007	MPPT: control	PQ 15 mg daily for 14 days	All individuals ≥ 5 years (except pregnant)	DOT (14 days for six rounds)	378,366–4,904,261 (94–98)	Pv	None	MPPT decreased PP considerably but failed to interrupt transmission
		Total duration: 6 years						

API = annual parasite index; CQ = chloroquine; DOT = directly observed treatment; IRS = indoor residual spraying; MDA = mass drug administration; MPPT = mass primaquine prophylactic treatment; ND = not described; Pf = *Plasmodium falciparum*; Pm = *Plasmodium malariae*; PP = parasite prevalence; PQ = primaquine; Pv = *Plasmodium vivax*; PYR = pyrimethamine.

**Table 3:** Examples of primaquine-containing MDA drug regimens

Study author Country Years	Drug regimen	G6PD considerations	Adverse events	Outcomes at conclusion of intervention
Primaquine to target *P. vivax* malaria (hypnozoites)				
Singh^30^ India 1962–1964	Total PQ dose = 75 mg	G6PD-deficient patients treated	None reported	Pv transmission suppressed during study period; incidence decreased from 0.98 to 0.006 cases/1,000 persons/month, maintained over one year
	PQ 15 mg/day for 5 days + CQ 600 mg single dose for four rounds; first round treated everyone; subsequent rounds targeted only febrile cases and their contacts			
WHO^7^ Nissan, Papua New Guinea 1962	Total PQ dose = 360–720 mg	G6PD-deficient patients treated; GdA^−1^ deficiency prevalence on Nissan = 30%	Hemoglobin levels in deficient patients checked weekly; weeks 1 and 2 did not drop below 2 g%, rose by week 3; at end of 8 weeks, about 1 g% higher than at start of MDA	Pf eliminated from Nissan; Pv reduced to low level but not eliminated due to presence of PQ-tolerant Chesson-like strains
	PQ 45–60 mg given by local residents as DOT weekly for 8–12 weeks			
Kondrashin^8^ Afghanistan, Azerbaijan, DPRK, Tajikistan 1971–2007	Total PQ dose = 210 mg	G6PD-deficient patients treated, with close monitoring	Severe side effects related to G6PD deficiency (i.e., red or black urine) did not exceed 1%; minor side effects did not exceed 4%	Considerably reduced Pv malaria where alternate forms of malaria control were unavailable
	PQ 15 mg given as DOT daily for 14 days in seasonal settings, either before or after transmission season			
Hsiang^9^ China 1973–1983	Total PQ dose = 180 mg	G6PD-deficient patients treated	Not systematically monitored; 49 cases of acute hemolysis reported in five studies that identified severe adverse events in deficient patients	Seasonal MDA administered to almost 30 million people, malaria incidence decreased by 56.7% (1973–1976) and by 12.4% (1976–1983)
	PQ 22.5 mg daily for 8 days and PYR 50 mg daily for 2 days administered to entire villages in the spring, prior to transmission season			
Liu^19^ China 1981–1985	Total PQ dose = 180 mg	G6PD-deficient patients treated	None reported	Incidence of Pv decreased; prevalence maintained at 0% for three years of post-MDA follow-up
	During low transmission season: CQ 1,200 mg total + PQ 180 mg total over 8 days; During high transmission season: CQ 300 mg + PQ 30 mg twice per month			
Kaneko^14^ Aneityum, Vanuatu 1991	Total PQ dose = 360 mg	G6PD-deficiency not detected on Aneityum	None reported	Sustained interruption of malaria transmission
	CQ 600 mg + SP 1,500 mg/75 mg + PQ 45 mg once in weeks 1, 5, 9; CQ 300 mg + PQ 45 mg once in weeks 2–4 and 6–8			
Hsiang^9^ China 2000–2009	Total PQ dose = 180 mg	G6PD-deficient patients treated	Five subjects in 2003 and two in 2007; some experienced hemolysis	Malaria incidence decreased by 14–44% in the two counties where MDA was conducted
	CQ 400 mg daily for 3 days + PQ 22.5 mg daily for 8 days, targeted to household members and neighbors of index cases in the spring			
Primaquine to target *P. falciparum* malaria (gametocytes)				
Hii^38^ Malaysia 1984–1985	Total PQ dose = 60 mg	G6PD-deficient patients treated	None reported	Pf prevalence temporarily decreased for 2 months after intervention; Pv prevalence did not change
	SP 1,430–70 mg + PQ 30 mg once per month for 2 months			
Doi^39^ Indonesia 1987–1989	Total PQ dose = 84–120 mg SP 25–30 mg/kg - 1.25–1.5 mg/kg single dose + PQ 0.7–1.0 mg/kg once per week for 2 weeks	G6PD-deficient patients treated	None reported	Pf prevalence declined from 14% to 1% eight months after MDA
Song^13^ Cambodia 2003–2004	Total PQ dose = 162 mg	G6PD-deficient patients treated	None reported	Parasite prevalence rate declined from 52.3% to 2.6% after 3 years
	Artemisinin 125 mg + PIP 750 mg daily for 2 days + PQ 9 mg every 10 days for 6 months			
Li (unpublished) Moheli Island, Comoros 2007	Total PQ dose = 108 mg	G6PD-deficient patients treated	None reported	Parasite prevalence rate declined from 21.6% to 0.86% after 18 months
	Artemisinin 125 mg + PIP 750 mg daily for 2 days, in 2 monthly rounds + PQ 9 mg every 10 days for 4 months			
Mahidol-Oxford Research Unit Protocol^40^ Cambodia 2013–2014	Total PQ dose = 45 mg	G6PD-deficient patients treated	Results not yet available	Results not yet available
	DHA 40 mg + PIP 320 mg daily as DOT for 3 days + PQ 0.25 mg/kg on day 1; regimen given monthly for 3 months			

CQ = chloroquine; DHA = dihydroartemisinin; DOT = directly observed treatment; G6PD = glucose-6-phosphate dehydrogenase; MDA = mass drug administration; MPPT = mass primaquine prophylactic treatment; Pf = *Plasmodium falciparum*; PIP = piperaquine; PQ = primaquine; Pv = *Plasmodium vivax*; PYR = pyrimethamine; SP = sulfadoxine-pyrimethamine.
