# Supplementary material for: Review of Mass Drug Administration for Malaria and Its Operational Challenges
Source: Am J Trop Med Hyg. 2015 Jul 8;93(1):125–34. doi: 10.4269/ajtmh.14-0254 (PMC4497884; doi:10.4269/ajtmh.14-0254)
Supplement: Supplementary file 1 [file SD5.pdf]

SUPPLEMENTAL APPENDIX A: 240 STUDIES ASSESSED FOR INCLUSION

The 72 studies excluded from this review are in *italics* and the 31 studies assessed as part of the Cochrane Review are in **bold** (note: Paik 1974 was included in the Cochrane Review as two separate studies, bringing the total number of assessed studies to 32)

1. Abraham AC, Samuels RD, 1944. Epidemiology of malaria in the Nizamsagar Ayacut area, Nizamabad District, Hyderabad State. *J Malar Inst India 5:* 305–318.
2. Afridi MK, Rahim A, 1959. Further observation on the interruption of malaria transmission with single dose of pyrimethamine (daraprim). *Riv Parassitol 20:* 229–242.
3. Ahorlu CK, Koram KA, Seake-Kwawu A, Weiss MG, 2011. Two-year evaluation of Intermittent Preventive Treatment for Children (IPTc) combined with timely home treatment for malaria control in Ghana. *Malar J 10:* 127.
4. Ahorlu CK, Koram KA, Seakey AK, Weiss MG, 2009. Effectiveness of combined intermittent preventive treatment for children and timely home treatment for malaria control. *Malar J 8:* 292.
5. *Aikins MK, Pickering H, Alonso PL, D'Alessandro U, Lindsay SW, Todd J, Greenwood BM, 1993. A malaria control trial using insecticide-treated bed nets and targeted chemoprophylaxis in a rural area of The Gambia, West Africa: 4. Perceptions of the causes of malaria and of its treatment and prevention in the study area. Trans R Soc Trop Med Hyg 87: 25–30.*
6. *Alicata JE, Dajani SW, 1955. Observation of pyrimethamine (daraprim) as a suppressant of malaria in a small village in Jordan. Am J Trop Med Hyg 4: 1006–1008.*
7. *Aliev S, Saparova N, 2001. The current malaria situation and its control in Tajikistan. Med Parazitol (Mosk) 1: 35–37.*
8. Aliev SP, 2000. Malaria in the Republic of Tadjikistan. *Med Parazitol (Mosk) 2:* 27–29.
9. Allen SJ, Otoo LN, Cooke GA, O'Donnell A, Greenwood BM, 1990. Sensitivity of *Plasmodium falciparum* to Maloprim after five years of targeted chemoprophylaxis in a rural area of The Gambia. *Trans R Soc Trop Med Hyg 84:* 666–667.
10. *Alonso PL, Lindsay SW, Armstrong Schellenberg JRM, Keita K, Gomez P, Shenton FC, Hill AG, David PH, Fegan G, Cham K, Greenwood BM, 1993. A malaria control trial using insecticide-treated bed nets and targeted chemoprophylaxis in a rural area of The Gambia, West Africa: 6. The impact of the interventions on mortality and morbidity from malaria. Trans R Soc Trop Med Hyg 87: 37–44.*
11. *Alonso PL, Lindsay SW, Armstrong Schellenberg JRM, Konteh M, Keita K, Marshall C, Phillips A, Cham K, Greenwood BM, 1993. A malaria control trial using insecticide-treated bed nets and targeted chemoprophylaxis in a rural area of The Gambia, West Africa: 5. Design and implementation of the trial. Trans R Soc Trop Med Hyg 87: 31–36.*
12. *Alving AS, Arnold J, Robinson DH, 1952. Mass therapy of subclinical vivax malaria with primaquine. JAMA 149: 1558–1562.*
13. Amangeldiev KA, 2001. The current malaria situation in Turkmenistan. *Med Parazitol (Mosk) 1:* 37–39.
14. *Archambeault CP, 1954. Mass antimalarial therapy in veterans returning from Korea. JAMA 154: 1411–1415.*
15. **Archibald HM, 1960. Field trials of mass administration of antimalarial drugs in northern Nigeria. *Bull World Health Org Mal 262:* 1–11.**
16. Archibald HM, Bruce-Chwatt LJ, 1956. Suppression of Malaria with pyrimethamine in Nigerian schoolchildren. *Bull World Health Organ 15:* 775–784.
17. Babione RW, 1966. Epidemiology of malaria eradication in central America: a study of technical problems. *Am J Public Health 56:* 76–90.
18. Banerjea R, 1949. The control of malaria in a rural area of West Bengal. *Indian J Malariol 3:* 371–386.
19. Barber MA, Rice JB, Brown JY, 1932. Malaria studies on the firestone rubber plantation in Liberia, West Africa. *Am J Hyg 15:* 601–633.
20. *Barger B, Maiga H, Traore OB, Tekete M, Tembine I, Dara A, Traore ZI, Gantt S, Doumbo OK, Djimde AA, 2009. Intermittent preventive treatment using artemisinin-based combination therapy reduces malaria morbidity among school-aged children in Mali. Trop Med Int Health 14: 784–791.*
21. Baukapur SN, Babu CJ, 1984. A focal outbreak of malaria in Valsad district, Gujarat state. *J Commun Dis 16:* 268–272.
22. Berberian DA, Dennis EW, 1948. Field experiments with chloroquine diphosphate. *Am J Trop Med 28:* 755–776.
23. Berny P, Nicolas L, 1936. Prophylaxis of malaria with quinacrine and rhodoquine in French Guiana. *Bull Soc Pathol Exot 29:* 870–872.
24. Bloch M, 1982. Teachings of the antimalarial campaign in El Salvador, central America. *Rev Inst Invest Med 11:* 119–124.
25. *Bojang K, Akor F, Bittaye O, Conway D, Bottomley C, Milligan P, Greenwood B, 2010. A randomised trial to compare the safety, tolerability and efficacy of three drug combinations for intermittent preventive treatment in children. PLoS One 5: e11225.*
26. *Bojang KA, Sesay S, Sowe M, Conway D, Milligan P, Greenwood B, 2009. A study of intermittent preventive treatment and home based management of malaria in a rural area of The Gambia. Am J Trop Med Hyg 81: 145.*
27. Bojang KA, Akor F, Conteh L, Webb E, Bittaye O, Conway DJ, Jasseh M, Wiseman V, Milligan PJ, Greenwood B, 2011. Two strategies for the delivery of IPTc in an area of seasonal malaria transmission in The Gambia: a randomised controlled trial. *PLoS Med 8:* e1000409.
28. *Boulanger D, Sarr JB, Fillol F, Cisse B, Sokhna C, Riveau G, Bork Simondon K, Trape JF, Greenwood B, Simondon F, Remoue F, 2009. Intermittent preventive treatment of malaria decreases the anti-Plasmodium schizont antibody response of Senegalese children. Trop Med Int Health 14: 31.*
29. *Boulanger D, Sarr JB, Fillol F, Sokhna C, Cisse B, Schacht AM, Trape JF, Riveau G, Simondon F, Greenwood B, Remoue F, 2010. Immunological consequences of intermittent preventive treatment against malaria in Senegalese preschool children. Malar J 9: 363.*
30. Brink CJH, 1958. Malaria control in the northern Transvaal. *S Afr Med J 32:* 800–809.
31. *Bruce-Chwatt LJ, 1983. Mass drug administration for control of malaria. Lancet 2: 688.*
32. Butler FA, 1943. Malaria control program on a South Pacific base. *U S Nav Med Bull 41:* 1603–1612.
33. *Cáceres Garcia JL, 2004. Eficacia de la cura radical masiva en la incidencia malárica del Municipio Mariño, Estado Sucre. Bol malariol salud ambient 44: 45–49.*
34. Cáceres Garcia JL, 2004. Estado Sucre: El éxito antimalárico de Venezuela en el año 2003. *Bol malariol salud ambient 44:* 51–55.
35. Cáceres Garcia JL, Pizzo N, Vela FA, Perez W, Rojas JG, Mora JD, Sanchez E, Paez E, Butron L, Rubio N, Maldonado A, 2005. Impacto de la Cura Radical Masiva sobre la incidencia malárica del estado Sucre, Venezuela. *Bol malariol salud ambient 45:* 27–36.
36. **Cáceres Garcia JL, 2008. Malaria antes y después de la cura radical masiva en el Estado Sucre, Venezuela. *Bol malariol salud ambient 48:* 83–90.**
37. Canet J, 1936. Prevention of malaria by the administration of synthetic drugs in the rubber plantations. *Prophylaxie Collective par Medicaments Synthetiques Sur Les Plantations des Terres-rouges* (1934–1936). 533–559.
38. Canet J, 1939. Results of four years mass prophylaxis with synthetic drugs in plantations in north Cochin-China. *Bull Soc Pathol Exot 32:* 58–69.
39. Canet J, 1949. First trials in southern Indo-China of mass prophylaxis of malaria with Nivaquine B (Resoquine) and with paludrine. *Bull Soc Pathol Exot 42:* 165–168.
40. Canet J, 1953. Proguanil resistance during mass prophylaxis of hyperendemic *P. falciparum* malaria in Indo-China. *Bull Soc Pathol Exot 46:* 230–245.
41. Canet J, Farinaud E, 1952. First trials of mass prophylaxis of malaria in Indo-China by daraprim. *Bull Soc Pathol Exot 45:* 645–652.
42. Capponi M, 1953. Note on malaria in Douala. *Med Trop 13:* 361–364.
43. **Cavalie P, Mouchet J, 1962. Les Campagnes Experimentales d'eradication du paludisme dans le nord de la Republique du Cameroun. *Med Trop 22:* 95–118.**

44. *Celli A, 1914. Malaria in Italy in 1912. Ann Ig 24: 177–243.*
45. *Charles LJ, 1958. Comparative assessment of chloroquine and amodiaquine as malaria suppressives in Nigeria. Ann Trop Med Parasitol 52: 55–67.*
46. Charles LJ, Van Der Kaay HJ, Vincke IH, Brady J, 1962. The appearance of pyrimethamine resistance in *Plasmodium falciparum* following self-medication by a rural community in Ghana. *Bull World Health Organ 26:* 103–108.
47. *Charles LJ, 1960. Aftermath of a field trial in self-administered pyrimethamine in a Ghanaian community: the appearance of P. falciparum resistance. Bull World Health Org Mal 260: 1–11.*
48. Chaudhuri RN, Poti SJ, 1950. Suppressive treatment of malaria. *Indian J Malariol 4:* 115–133.
49. *Chen W, Wu K, Lin M, Tang L, Gu Z, Wang S, Lan C, Lan X, Li H, Huang M, Chen X, Sheng H, 1999. A pilot study on malaria control by using a new strategy of combining strengthening infection source treatment and health education in mountainous areas of Hainan province. Chinese J Parasitol Parasit Dis 17: 1–4.*
50. Cisse B, Cairns M, Faye E, NDiaye O, Faye B, Cames C, Cheng Y, NDiaye M, Lo AC, Simondon K, Trape JF, Faye O, NDiaye JL, Gaye O, Greenwood B, Milligan P, 2009. Randomized trial of piperaquine with sulfadoxine-pyrimethamine or dihydroartemisinin for malaria intermittent preventive treatment in children. *PLoS One 4:* e7164.
51. *Cisse B, Sokhna C, Boulanger D, Milet J, Ba EH, Richardson K, Hallett R, Sutherland C, Simondon K, Simondon F, Alexander N, Gaye O, Targett G, Lines J, Greenwood B, Trape JF, 2006. Seasonal intermittent preventive treatment with artesunate and sulfadoxine-pyrimethamine for prevention of malaria in Senegalese children: a randomised, placebo-controlled, double-blind trial. Lancet 367: 659–667.*
52. Ciuca M, Balteanu I, Alexa I, 1937. Experimental control of malaria with synthetic drugs. *Arch Roum Pathol Exp Microbiol 10:* 295–306.
53. Clark HC, Komp WHW, Jobbins DM, 1942. A tenth year's observations on malaria in Panama, with reference to the occurrence of variations in the parasite index, during continued treatment with atabrine and plasmochine. *Am J Trop Med Hyg 22:* 191–216.
54. Clarke SE, Jukes MCH, Njagi JK, Khasakhala L, Cundill B, Otido J, Crudder C, Estambale BBA, Brooker S, 2008. Effect of intermittent preventive treatment of malaria on health and education in schoolchildren: a cluster-randomised, double-blind, placebo-controlled trial. *Lancet 372:* 127–138.
55. Clyde DF, 1961. Malaria control in Tanganyika under the German administration, Part I. *East Afr Med J 38:* 27–42.
56. Clyde DF, 1961. Malaria control in Tanganyika under the German administration, Part II: mass chemoprophylaxis in Dar es Salaam. *East Afr Med J 38:* 69–82.
57. Clyde DF, 1962. Mass administration of an antimalarial drug combining 4-aminoquinoline and 8-aminoquinoline in Tanganyika. *Bull World Health Organ 27:* 203–212.
58. Clyde DF, Webbe G, Shute GT, 1958. Single dose pyrimethamine treatment of Africans during a malaria epidemic in Tanganyika. *East Afr Med J 35:* 23–29.
59. **Comer RD, Young MD, Johnson CM, Babione RW, 1971. Mass drug trial of pyrimethamine and primaquine for the eradication of malaria in Sambu, Republic of Panama. *Bol Of Sanit Panam (Engl) 70:* 226–233.**
60. *Cornille Brogger R, Mathews HM, Storey J, Ashkar TS, Brogger S, Molineaux L, 1978. Changing patterns in the humoral immune response to malaria before, during and after the application of control measures: a longitudinal study in the West African savanna. Bull World Health Organ 56: 579–600.*
61. Coutinho Da Costa F, Viana De Meira L, 1962. Malaria and anti-malarial campaign in Bissau. *Bol Cult Guine Portuguesa 17:* 119–165.
62. *D'Anfreville De La Salle L, 1930. A method of dealing with malaria in Morocco. Bull Soc Pathol Exot 23: 53–58.*
63. *Danquah I, Dietz E, Zanger P, Reither K, Ziniel P, Bienzle U, Mockenhaupt FP, 2009. Reduced efficacy of intermittent preventive treatment of malaria in malnourished children. Antimicrob Agents Chemother 53: 1753–1759.*
64. Dapeng L, Leyuan S, Xili L, Xiance Y, 1996. A successful control programme for falciparum malaria in Xinyang, China. *Trans R Soc Trop Med Hyg 90:* 100–102.
65. *De Martin S, von Seidlein L, Deen JL, Pinder M, Walraven G, Greenwood B, 2001. Community perceptions of a mass administration of an antimalarial drug combination in The Gambia. Trop Med Int Health 6: 442–448.*
66. De Mello IF, 1938. Anti-malaria measures in rural areas of Portuguese India. *Riv Malariol 17:* 208–224.
67. **De Zulueta J, Kafuko GW, Cullen JR, Pedersen CK, 1961. The results of the first year of a malaria eradication pilot project in northern Kigezi (Uganda). *East Afr Med J 38:* 1–26.**
68. **De Zulueta J, Kafuko GW, McCrae AWR, Cullen JR, Pedersen CK, Wasswa DFB, 1964. A malaria eradication experiment in the highlands of Kigezi (Uganda). *East Afr Med J 41:* 102–120.**
69. *Decourt P, 1935. Mixed drug prophylaxis in malaria. Bull Soc Pathol Exot 28: 255–261.*
70. Decourt P, Dupoux R, Belfort, Henry C, 1936. Mass prophylaxis of malaria in Tunisia. *Bull Soc Pathol Exot 29:* 487–493.
71. *Delmont J, Ranque P, Balique H, Tounkara A, Soula G, Quilici M, Pene P, 1981. Influence of antimalarial chemoprophylaxis on the health status of a rural community in West Africa: preliminary results. Bull Soc Pathol Exot 74: 600–610.*
72. Desowitz RS, Spark RA, 1987. Malaria in the Maprik area of the Sepik region, Papua New Guinea: 1957–1984. *Trans R Soc Trop Med Hyg 81:* 175–176.
73. Diallo S, Coulibaly A, Konate M, Samba O, 1977. Chloroquine prophylaxis and the prevalence of malaria. *Med Afr Noire 24:* 117–125.
74. Diallo S, Diouf F, Bah IB, N'Dir O, Victorius A, 1983. Clinical consequences of chloroquine prophylaxis and of its discontinuation in a hyperendemic malarial region. *Dakar Méd 28:* 43–65.
75. *Dicko A, Sagara I, Sissoko MS, Guindo O, Diallo AI, Kone M, Toure OB, Sacko M, Doumbo OK, 2008. Impact of intermittent preventive treatment with sulphadoxine-pyrimethamine targeting the transmission season on the incidence of clinical malaria in children in Mali. Malar J 7: 123.*
76. *Dicko A, Diallo AI, Tembine I, Dicko Y, Dara N, Sidibe Y, Santara G, Diawara H, Conare T, Djimde A, Chandramohan D, Cousens S, Milligan PJ, Diallo DA, Doumbo OK, Greenwood B, 2011. Intermittent preventive treatment of malaria provides substantial protection against malaria in children already protected by an insecticide-treated bednet in Mali: a randomised, double-blind, placebo-controlled trial. PLoS Med 8: e1000407.*
77. Dixon DS, 1950. Paludrine (Proguanil) as a malarial prophylactic amongst African labour in Kenya. *East Afr Med J 27:* 127–130.
78. Doi H, Kaneko A, Panjaitan W, Ishii A, 1989. Chemotherapeutic malaria control operation by single dose of Fansidar plus primaquine in North Sumatra, Indonesia. *Southeast Asian J Trop Med Public Health 20:* 341–349.
79. Dola SK, 1974. Mass drug administration as a supplementary attack measure in malaria eradication programme. *East Afr Med J 51:* 529–531.
80. Doucet G, 1947. Preliminary note on the use of S.N. 7618 in a hyperendemic malarial locality. *Ann Soc Belg Med Trop 27:* 341–346.
81. *Downs WG, 1946. Results in an infantry regiment of several plans of treatment for vivax malaria. Am J Trop Med 26: 67–86.*
82. Dupoux R, Barthas R, Antoine A, Garali MT, 1939. Recent results of experiment in collective antimalarial prophylaxis in Tunis. *Bull Acad Med 121:* 591–595.
83. Dupoux R, Marini C, Barthas R, 1937. Mass prophylaxis of malaria in Tunis. *Bull Acad Med 118:* 368–372.
84. Edeson JFB, Wharton RH, Wilson T, Reid JA, 1957. An experiment in the control of rural malaria in Malaya. *Med J Malaya 12:* 319–347.
85. **Escudie A, Hamon J, Schneider J, 1962. Results of mass antimalarial chemoprophylaxis with a combination of 4-aminoquinoline and 8-aminoquinoline under rural African conditions in the region of Bobo-Dioulasso (Upper Volta)**

1960: comparative study in a zone treated with DDT and outside this zone. *Med Trop 22:* 268–305.

86. Farinaud M, 1934. Testing malaria prophylaxis in infants in Tri-Cu. *Bull Soc Pathol Exot 27:* 568–575.
87. Farinaud ME, Choumara R, 1950. Malarial infestation and demography of the mountain population of southern Indo-China (P.M.S.I.). Part 1: malaria among the P.M.S.I., chemoprophylaxis and DDT dusting. *Bull Econ Indochine 22:* 5–22.
88. **Gabaldon A, Guerrero L, 1959. An attempt to eradicate malaria by the weekly administration of pyrimethamine in areas of out-of-doors transmission in Venezuela. *Am J Trop Med Hyg 8:* 433–439.**
89. *Garfield R, 1999. Malaria control in Nicaragua: social and political influences on disease transmission and control activities. Lancet 354: 414–418.*
90. *Garfield RM, Vermund SH, 1984. Malaria in Nicaragua: an update. Lancet 1: 1125.*
91. **Garfield RM, Vermund SH, 1983. Changes in malaria incidence after mass drug administration in Nicaragua. *Lancet 2:* 500–503.**
92. *Garfield RM, Vermund SH, 1986. Health education and community participation in mass drug administration for malaria in Nicaragua. Soc Sci Med 22: 869–877.*
93. **Gaud J, Houel G, 1953. Individual and mass treatment of malaria by a single dose of flavoquine. *Bull Soc Pathol Exot 46:* 565–571.**
94. Gaud J, Schneider J, Mechali D, 1949. Comparative efficacy of nivaquine and chloriguane in mass prophylaxis of malaria. *Bull Inst Hyg Maroc 9:* 121–129.
95. Gilroy AB, 1952. Proguanil-resistant *Plasmodium falciparum* in Assam. *Ann Trop Med Parasitol 46:* 121–126.
96. Gomez Mendoza I, 1960. Observations on the programme for the employment of antimalarial drugs in the malaria eradication campaign in Venezuela. *CNEP Bol 4:* 74–81.
97. Gribben G, 1933. Mass treatment with plasmoquine. *BMJ 3802:* 919–920.
98. Gruer N, Ousset JH, Lopez Manan CE, 1962. Special problems in the malaria eradication campaign. *An Inst Nac Microbiol 1:* 127–131.
99. *Gunther CEM, 1951. Proguanil hydrochloride (paludrine) in the prevention and treatment of malaria in New Guinea. Trans R Soc Trop Med Hyg 44: 473–478.*
100. *Gunther CEM, Fraser NM, Wright WG, 1952. Proguanil and malaria among non-tolerant New Guinea natives. Trans R Soc Trop Med Hyg 46: 185–190.*
101. *Gusmao HH, Juarez E, 1970. A trial of CI-564 (Dapolar), a repository antimalarial for prophylaxis in Amapá, Brazil. Am J Trop Med Hyg 19: 394–400.*
102. Han ET, Lee DH, Park KD, Seok WS, Kim YS, Tsuboi T, Shin EH, Chai JY, 2006. Reemerging vivax malaria: changing patterns of annual incidence and control programs in the Republic of Korea. *Korean J Parasitol 44:* 285–294.
103. Harwin RM, 1973. A field trial of the effectiveness of cycloguanil pamoate in Rhodesia. *Cent Afr J Med 19:* 9–12.
104. Henderson LH, 1934. Prophylaxis of malaria in the Sudan, with special reference to the use of plasmoquine. *Trans R Soc Trop Med Hyg 28:* 157–164.
105. **Hii JLK, Vun YS, Chin KF, Chua R, Tambakau S, Binisol ES, Fernandez E, Singh N, Chan MKC, 1987. The influence of permethrin-impregnated bednets and mass drug administration on the incidence of *Plasmodium falciparum* malaria in children in Sabah, Malaysia. *Med Vet Entomol 1:* 397–407.**
106. Ho C, 1965. Studies on malaria in new China. *Chin Med J 84:* 491–497.
107. **Houel G, 1954. Treatment of epidemic-malaria with a single dose of pyrimethamine. *Bull Soc Pathol Exot 47:* 262–264.**
108. Houel G, Van Goor WT, 1954. Chemoprophylaxis of malaria with monthly doses of chloroquine and amodiaquine. *Bull Soc Pathol Exot 47:* 254–260.
109. Huehne WH, 1971. Experience with an insecticide/drug combination and observations on suppressive chloroquine/pyrimethamine treatment. *J Trop Med Hyg 74:* 110–116.
110. Janssens PG, Verstraete N, Sieniawski J, 1950. Trials of collective antimalaria drug prophylaxis among children of mine workers at Kilo. *Ann Soc Belg Med Trop 30:* 257–286.
111. Joncour G, 1956. La Lutte Contre Le Paludisme A Madagascar. *Bull World Health Organ 15:* 711–723.
112. **Jones SA, 1954. Resistance of *P. falciparum* and *P. malariae* to pyrimethamine (daraprim) following mass treatment with this drug: a preliminary note. *East Afr Med J 31:* 47–49.**
113. **Jones SA, 1958. Mass treatment with pyrimethamine: a study of resistance and cross resistance resulting from a field trial in the hyperendemic malarious area of Makueni, Kenya, September 1952–September 1953. *Trans R Soc Trop Med Hyg 52:* 547–561.**
114. *Kaneko A, Taleo GK, Rieckmann KH, 1994. Island malaria control in eastern Melanesia: 1. Malaria eliminated from a small island by 9-week mass drug administration and impregnated bednets. Kisechugaku Zasshi 43: 358–370.*
115. *Kaneko A, 2010. A community-directed strategy for sustainable malaria elimination on islands: short-term MDA integrated with ITNs and robust surveillance. Acta Trop 114: 177–183.*
116. **Kaneko A, Taleo G, Kalkoa M, Yamar S, Kobayakawa T, Björkman A, 2000. Malaria eradication on islands. *Lancet 356:* 1560–1564.**
117. Karimov SS, Kadamov DS, Murodova NK, 2008. The current malaria situation in Tadjikistan. *Med Parazitol (Mosk) 1*: 33–36.
118. Kingsbury AN, Amies CR, 1931. A field experiment on the value of plasmoquine in the prophylaxis of malaria. *Trans R Soc Trop Med Hyg 25:* 159–172.
119. **Kligler IJ, Mer G, 1931. Periodic intermittent treatment with chinoplasmine as a measure of malaria control in a hyperendemic area. *Riv Malariol 10:* 425–438.**
120. Klopper S, 1949. The suppressive action of paludrine in benign tertian (vivax) malaria. *Doc Neerl Indones Morbis Trop 1:* 50–54.
121. Komp WHW, Clark HC, 1935. A fourth year's observations on malaria in Panama, with reference to control with atabrine and plasmochin. *Am J Trop Med 15:* 131–154.
122. *Konaté AT, Yaro JB, Ouédraogo AZ, Diarra A, Gansané A, Soulama I, Kangoyé DT, Kaboré Y, Ouédraogo E, Ouédraogo A, Tiono AB, Ouédraogo IN, Chandramohan D, Cousens S, Milligan PJ, Sirima SB, Greenwood B, Diallo DA, 2011. Intermittent preventive treatment of malaria provides substantial protection against malaria in children already protected by an insecticide-treated bednet in Burkina Faso: a randomised, double-blind, placebo-controlled trial. PLoS Med 8: e1000408.*
123. **Kondrashin AV, Sanyal MC, 1985. Mass drug administration in Andhra Pradesh in areas under *Plasmodium falciparum* containment programme. *J Commun Dis 17:* 293–299.**
124. *Kweku M, Liu D, Adjuik M, Binka F, Seidu M, Greenwood B, Chandramohan D, 2008. Seasonal intermittent preventive treatment for the prevention of anaemia and malaria in Ghanaian children: a randomized, placebo controlled trial. PLoS One 3: e4000.*
125. Kweku M, Webster J, Adjuik M, Abudey S, Greenwood B, Chandramohan D, 2009. Options for the delivery of intermittent preventive treatment for malaria to children: a community randomised trial. *PLoS One 4:* e7256.
126. Lacroix M, Mazzuca M, Bonnet M, 1952. Proguanil and malaria prophylaxis in two Algerian villages. *Bull Soc Pathol Exot 45:* 460–464.
127. Lahon H, De Smet M, Boets L, 1960. Results of 5 years of mass chemoprophylaxis with pyrimethamine in Yangambi, Congo. *Ann Soc Belg Med Trop 40:* 651–673.
128. Laing ABG, 1970. Malaria suppression with fortnightly doses of pyrimethamine with sulfadoxine in the Gambia. *Bull World Health Organ 43:* 513–520.
129. *Laing ABG, 1984. The impact of malaria chemoprophylaxis in Africa with special reference to Madagascar, Cameroon, and Senegal. Bull World Health Organ 62 (Suppl): 41–48.*
130. Lakshmanacharyulu T, Guha AK, Kache SR, 1968. Control of malaria epidemics in a river valley project. *Bull Ind Soc Malar Comm Dis 5:* 94–105.
131. Levenson ED, Fastorskaya EI, Khovanskaya AI, Duk-Hanina NN, 1943. Experiences in the control of a malarial focus in the north (Arehangel Région) by mass chemoprophylaxis and systematic treatment of malaria patients. *Med Parazitol (Mosk) 12:* 23–38.

132. *Liljander A, Chandramohan D, Kweku M, Olsson D, Montgomery SM, Greenwood B, Farnert A, 2010. Influences of intermittent preventive treatment and persistent multiclonal Plasmodium falciparum infections on clinical malaria risk. PLoS One 5: e13649.*
133. Liu Y, Wu K, Jia J, Jiang W, Wang K, Pan J, He J, Luo M, Zhang J, Zhang Y, Jiang B, Teng M, Liu J, Shao S, Wang Y, Zhang F, Zhang P, Zhou S, Wu X, Hu Z, Geng D, Wang Y, Li D, 1986. Integrated approach in malaria control including environmental management to reduce man-mosquito contact and reduction of infection source in Huanghuai Plain. *J Parasitol Parasit Dis 4:* 246–250.
134. Lysenko AY, 1960. Use of quinocide in treatment and prophylaxis of vivax malaria. *Bull World Health Organ 22:* 641–662.
135. *Miller MJ, 1955. Suppression of malaria by monthly drug administration. Am J Trop Med Hyg 4: 790–799.*
136. MacCormack CP, Lwihula G, 1983. Failure to participate in a malaria chemosuppression programme: North Mara, Tanzania. *J Trop Med Hyg 86:* 99–107.
137. Mackerras MJ, Sandars DF, 1954. *Malaria in the Torres Straits Islands*. South Pacific Commission Technical Paper No. 68. 1–27.
138. *Maiga H, Barger B, Traore OB, Tekete M, Timbine A, Dara A, Traore ZI, Gantt S, Doumbo O, Djimde A, 2009. Intermittent preventive treatment using artemisinin-based combination therapy reduces malaria morbidity among school-aged children in Mali. Am J Trop Med Hyg 81 (Suppl): 42.*
139. 1934. Annotations: malaria in the army in India. *Lancet 223:* 802–803.
140. Mason J, Hobbs J, 1977. Malaria field studies in a high-incidence coastal area of El Salvador, C.A. *Bull Pan Am Health Organ 11:* 17–30.
141. Mason J, Hobbs JH, 1973. A study of the epidemiology of malaria in a high-incidence coastal area of El Salvador, C.A. *Rev Inst Invest Med 2:* 51–57.
142. Mastbaum O, 1957. Malaria control in Swaziland: some observations during the first year of partial discontinuation of insecticides. *J Trop Med Hyg 60:* 190–192.
143. *McGregor IA, Williams K, Walker GH, Rahman AK, 1966. Cycloguanil pamoate in the treatment and suppression of malaria in the Gambia, West Africa. BMJ 1: 695–701.*
144. Melik-Adamian SS, 1938. Acriquine in the mass treatment of malarious children. *Med Parazitol (Mosk) 7:* 178–191.
145. Mendez Galvan JF, Alvarado JG, Mora MG, Landa MP, Cabanillas RQ, 1984. Evaluation of alternative schemes of treatment for malaria control. *Salud Publica Mex 26:* 561–572.
146. Mercier S, 1952. Epidemiological and demographic results of malaria control by residual spraying in Tananarive in 1950. *Rev Palud Med Trop 92:* 21–31.
147. Merle F, Maillot L, 1955. Vector control campaigns against malaria in Brazzaville. *Bull Soc Pathol Exot 48:* 242–269.
148. **Metselaar D, 1961. Seven years' malaria research and residual house spraying in Netherlands New Guinea. *Am J Trop Med Hyg 10:* 327–334.**
149. Mezincesco D, Cornelson DA, 1935. The prophylactic treatment of malaria with atebrin and with quinine. *Arch Roum Pathol Exp Microbiol 8:* 449–470.
150. *Molineaux L, Storey J, Cohen JE, Thomas A, 1980. A longitudinal study of human malaria in the West African savanna in the absence of control measures: relationships between different Plasmodium species, in particular P. falciparum and P. malariae. Am J Trop Med Hyg 29: 725–737.*
151. *Molineaux L, Cornille-Brogger R, Mathews HM, Storey J, 1978. Longitudinal serological study of malaria in infants in the West African savanna: comparisons in infants exposed to, or protected from, transmission from birth. Bull World Health Organ 56: 573–578.*
152. **Molineaux L, Gramiccia G, 1980. *The Garki Project: Research on the Epidemiology and Control of Malaria in the Sudan Savanna of West Africa*. Geneva, Switzerland: World Health Organization.**
153. Monteny VAR, 1960. Comparative efficacy of chloroquine and pyrimethamine as prophylactics against Malaria. *Ann Soc Belg Med Trop 40:* 511–516.
154. *Muhlens P, 1913. Report of a malaria expedition to Jerusalem. Zentralbl Bakteriol Parasit Infekt Hyg 69: 41–85.*
155. **Najera J, Shidrawi GR, Storey J, Lietaert PEA, 1973. Mass drug administration and DDT indoor-spraying as antimalarial measures in the northern savanna of Nigeria. *Malar Bull World Health Org 73:* 1–34.**
156. *Nakibuuka V, Ndeezi G, Nakiboneka D, Ndugwa CM, Tumwine JK, 2009. Presumptive treatment with sulphadoxine-pyrimethamine versus weekly chloroquine for malaria prophylaxis in children with sickle cell anaemia in Uganda: a randomized controlled trial. Malar J 8: 237.*
157. *Nankabirwa J, Cundill B, Clarke S, Kabatereine N, Rosenthal PJ, Dorsey G, Brooker S, Staedke SG, 2010. Efficacy, safety, and tolerability of three regimens for prevention of malaria: a randomized, placebo-controlled trial in Ugandan schoolchildren. PLoS One 5: e13438.*
158. Nave Rebollo O, Parada E, Guerra A, 1973. Malaria in El Salvador: control and eradication campaign analysis. *Rev Inst Invest Med 2:* 31–39.
159. Norman T, 1952. An investigation of the failure of proguanil prophylaxis. *Trans R Soc Trop Med Hyg 46:* 653–655.
160. *Ntab B, Cisse B, Boulanger D, Sokhna C, Targett G, Lines J, Alexander N, Trape JF, Simondon F, Greenwood BM, Simondon KB, 2007. Impact of intermittent preventive antimalarial treatment on the growth and nutritional status of preschool children in rural Senegal (West Africa). Am J Trop Med Hyg 77: 411–417.*
161. Omer AHS, 1978. Species prevalence of malaria in northern and southern Sudan, and control by mass chemoprophylaxis. *Am J Trop Med Hyg 27:* 858–863.
162. Onori E, 1972. Experience with mass drug administration as a supplementary attack measure in areas of vivax malaria. *Bull World Health Organ 47:* 543–548.
163. Ossi GT, 1967. An epidemic in the life of a malaria eradication programme. *Bull Endem Dis (Baghdad) 9:* 5–18.
164. *Ouédraogo A, Tiono AB, Diarra A, Nébié IO, Konaté AT, Sirima SB, 2010. The effects of a pre-season treatment with effective antimalarials on subsequent malaria morbidity in under five-year-old children living in high and seasonal malaria transmission area of Burkina Faso. Trop Med Int Health 15: 1315–1321.*
165. **Paik YH, Avery JG, 1974. Problem areas in the malaria eradication programme in the British Solomon Islands. *P N G Med J 17:* 61–67.**
166. Parrot L, Catanei A, Ambialet R, 1937. Comparative experiments in mass prophylaxis of malaria by means of quinine and of synthetic drugs (quinacrine and praequine). *Bull Health Org 6:* 683–684.
167. Parrot L, Catanei A, Collignon E, 1944. New trials of mass prophylaxis of malaria with synthetic drugs. *Arch Inst Pasteur Alger 22:* 179–246.
168. Parrot L, Catanei A, Collignon E, 1946. Further trials of mass prophylaxis of malaria with synthetic drugs. *Arch Inst Pasteur Alger 24:* 205–278.
169. Parrot L, Catanei A, Collignon E, Ambialet R, 1943. New trial of synthetic drugs for collective prophylaxis of malaria. *Arch Inst Pasteur Alger 21:* 131–179.
170. Peters W, 1962. A critical survey of the results of malaria-eradication and control programmes in the south-west Pacific. *Ann Trop Med Parasitol 56:* 20–32.
171. *Phillips MG, 1954. Malaria prophylaxis. BMJ 1: 155.*
172. Pikul J, Serguiev P, Tibourskaya N, 1934. Experiment on the prophylactic use of plasmocide in Daghestan with observations on the mosquito infection rate. *Med Parazitol (Mosk) 3:* 322–329.
173. Pribadi W, Muzaham F, Santoso T, Rasidi R, Rukmono B, Soeharto, 1986. The implementation of community participation in the control of malaria in rural Tanjung Pinang, Indonesia. *Southeast Asian J Trop Med Public Health 17:* 371–378.
174. Prokopenko LI, 1945. An analysis of the causes of the severe epidemic of malaria In 1942 in the Urgut district of the province of Samarkand and measures to prevent an Increase in malaria morbidity in 1943. *Med Parasitol 14:* 15–33.

175. Rachou RG, Lyons G, Moura-Lima M, Kerr JA, 1965. Synoptic epidemiológical studies of malaria in El Salvador. *Am J Trop Med Hyg 14:* 1–62.
176. Rafi SM, Shah IA, 1951. Paludrine as a causal prophylactic in hyperendemic areas. *Pak J Health 1:* 42–46.
177. Ray AP, 1948. Prophylactic use of paludrine in a tea estate. *Indian J Malariol 2:* 35–66.
178. Ricosse J, Bailly-Choumara H, Adam JP, Hamon J, 1959. Resultats d'une experimentation de chimioprophylaxie par la pyrimethamine dans la zone pilote de lutte antipaludique de Bobo-Dioulasso. *Bull Soc Pathol Exot 52:* 516–535.
179. Roberts JMD, 1956. Pyrimethamine (daraprim) in the control of epidemic malaria. J Trop Med Hyg 59: 201–208.
180. Roberts JMD, 1964. The control of epidemic malaria in the highlands of western Kenya. Part I: before the campaign. *J Trop Med Hyg 67:* 161–168.
181. Roberts JMD, 1964. The control of epidemic malaria in the highlands of western Kenya. Part II: the campaign. J Trop Med Hyg 67: 191–199.
182. Roberts JMD, 1964. The control of epidemic malaria in the highlands of western Kenya. Part III: after the campaign. J Trop Med Hyg 67: 230–237.
183. Robin C, Brochen L, 1946. Malaria in Dakar: results of the therapeutic and prophylactic administration of synthetic drugs in a native population. *Bull Med Afr-Occ Franc 3:* 97–108.
184. Rodríguez López MH, Elizondo EGL, Reyes AFB, Treviño CV, Bown DN, 1994. Control focal del paludismo: tratamiento focal usando quimioprofilaxis y rociado intradomiciliar con insecticida para el control del paludismo en el sur de México. *Gac Med Mex 130:* 313–319.
185. Rohner F, Zimmermann MB, Amon RJ, Vounatsou P, Tschannen AB, N'Goran EK, Nindjin C, Cacou MC, Te-Bonle MD, Aka H, Sess DE, Utzinger J, Hurrell RF, 2010. In a randomized controlled trial of iron fortification, anthelmintic treatment, and intermittent preventive treatment of malaria for anemia control in Ivorian children, only anthelmintic treatment shows modest benefit. J Nutr 140: 635–641.
186. Saarinen M, Iyambo N, Shinyafa L, Paajanen H, Indongo I, Thoren E, Carlstedt A, Fernanda M, Paajanen K, Rombo L, 1987. Mass proguanil prophylaxis. *Lancet 1:* 985–986.
187. Salako LA, Ajayi FO, Sowunmi A, Walker O, 1990. Malaria in Nigeria: a revisit. Ann Trop Med Parasitol 84: 435–445.
188. Salihu HM, Tchuinguem G, Ratard R, 2000. Effect of chloroquine prophylaxis on birthweight and malaria parasite load among pregnant women delivering in a regional hospital in Cameroon. West Indian Med J 49: 143–147.
189. Santos JB, Prata A, Wanssa E, 1993. Quimioprofilaxia da malária com mefloquina na Amazônia Brasileira. Rev Soc Bras Med Trop 26: 157–162.
190. Schliessmann DJ, Joseph VR, Solis M, Carmichael GT, 1973. Drainage and larviciding for control of a malaria focus in Haiti. *Mosq News 33:* 371–378.
191. Schneider J, Languillon J, Delas A, 1958. Association chloroquine-pyrimethamine dans la chimioprophylaxie du paludisme resultats apres 22 mois de traitement. *Bull Soc Pathol Exot 51:* 316–319.
192. Schneider J, Escudie A, Ouedraogo A, Sales P, 1962. Chimioprophylaxie du paludisme par distributions hebdomadaires de chloroquine ou d'une association chloroquine-primaquine-pyrimethamine. Bull Soc Pathol Exot 2: 280–290.
193. Schneider J, Dignat M, Voron, Sfar M, 1948. Mass prophylaxis of malaria with premaline in the Gabes area, May to November, 1946. *Bull Soc Pathol Exot 41:* 194–198.
194. Schneider J, Escudie A, Hamon J, 1961. Eradication of malaria and chemotherapy: results obtained with the association Amino-4 Quinoline + Amino-8 Quinoline in the pilot area of Bobo-Dioulasso (Upper Volta). *Bull Soc Pathol Exot 54:* 1012–1025.
195. Schneider J, Larabi M, Balti M, 1948. Mass prophylaxis of malaria with nivaquine: results of an experience in Ghardimaou, Tunisia. *Bull Soc Pathol Exot 41:* 188–194.
196. Seckinger DL, 1935. Atabrine and plasmochin in the treatment and control of malaria. *Am J Trop Med 15:* 631–649.
197. Sehgal JK, 1968. Progress of malaria eradication in Orissa State during 1965–66. *Bull Ind Soc Mal Com Dis 5:* 88–93.
198. Sergent E, Sergent E, 1913. Epidemiological and prophylactic studies of malaria. *Ann Inst Pasteur (Paris) 27:* 373–390.
199. Sesay S, Milligan P, Touray E, Sowe M, Webb EL, Greenwood BM, Bojang KA, 2011. A trial of intermittent preventive treatment and home-based management of malaria in a rural area of The Gambia. Malar J 10: 2.
200. Shanks GD, Barnett A, Edstein MD, Rieckmann KH, 1995. Effectiveness of doxycycline combined with primaquine for malaria prophylaxis. Med J Aust 162: 306–310.
201. Shanks GD, Edstein MD, Kereu RK, Spicer PE, Rieckmann KH, 1993. Postexposure administration of halofantrine for the prevention of malaria. Clin Infect Dis 17: 628–631.
202. Shanks GD, Edstein MD, Suriyamongkol V, Timsaad S, Webster HK, 1992. Malaria chemoprophylaxis using proguanil/dapsone combinations on the Thai-Cambodian border. Am J Trop Med Hyg 46: 643–648.
203. Shanks GD, Roessler P, Edstein MD, Rieckmann KH, 1995. Doxycycline for malaria prophylaxis in Australian soldiers deployed to United Nations missions in Somalia and Cambodia. Mil Med 160: 443–445.
204. Sheinker KP, 1945. An experiment in epidemiological chemical prophylaxis at a site of new construction in central Asia. *Med Parazitol (Mosk) 14:* 56–62.
205. Shekalaghe SA, Drakeley C, van den Bosch S, ter Braak R, van den Bijllaardt W, Mwanziva C, Semvua S, Masokoto A, Mosha F, Teelen K, Hermsen R, Okell L, Gosling R, Sauerwein R, Bousema T, 2011. A cluster-randomized trial of mass drug administration with a gametocytocidal drug combination to interrupt malaria transmission in a low endemic area in Tanzania. *Malar J 10:* 247.
206. Shekalaghe SA, ter Braak R, Daou M, Kavishe R, van den Bijllaardt W, van den Bosch S, Koenderink JB, Luty AJF, Whitty CJM, Drakeley C, Sauerwein RW, Bousema T, 2010. In Tanzania, hemolysis after a single dose of primaquine coadministered with an artemisinin is not restricted to glucose-6-phosphate dehydrogenase-deficient (G6PD A-) individuals. Antimicrob Agents Chemother 54: 1762–1768.
207. Simeons ATW, 1936. Mass treatment with injectable atebrin. Ind Med Gaz 71: 132–137.
208. Simeons ATW, 1938. Follow-up of a mass treatment with injectable atebrin. *Ind Med Gaz 73:* 713–715.
209. Singh J, Misra BG, Ray AP, 1953. Suppressive treatment with amodiaquin. *Ind J Malar 7:* 27–31.
210. Singh MV, Agarwala RS, Singh KN, 1968. Epidemiological study of focal outbreak of malaria in consolidation phase area and evaluation of remedial measures in Uttar Pradesh (India). *Bull Ind Soc Mal Com Dis 5:* 207–220.
211. Snowden FM, 2006. *The Conquest of Malaria: Italy, 1900–1962*. New Haven, CT: Yale University Press.
212. Sokhna C, Cisse B, Ba EH, Milligan P, Hallett R, Sutherland C, Gaye O, Boulanger D, Simondon K, Simondon F, Targett G, Lines J, Greenwood B, Trape JF, 2008. A trial of the efficacy, safety and impact on drug resistance of four drug regimens for seasonal intermittent preventive treatment for malaria in Senegalese children. PLoS One 3: e1471.
213. Song J, Socheat D, Tan B, Dara P, Deng C, Sokunthea S, Seila S, Ou F, Jian H, Li G, 2010. Rapid and effective malaria control in Cambodia through mass administration of artemisinin-piperaquine. *Malar J 9:* 57.
214. Sorel F, 1913. Hygiene in Bassam in 1912. *Bull Soc Pathol Exot 6:* 645–653.
215. Srivastava RS, 1950. Malaria control measures in the Tarai area under the Tarai colonization scheme, Kichha, District Naini Tal: September 1947 to December 1948: first report. *Indian J Malariol 4:* 151–165.
216. Strangways Dixon D, 1950. Paludrine (Proguanil) as a malarial prophylactic amongst African labour in Kenya. *East Afr Med J 28:* 126–130.
217. Strickland GT, Khaliq AA, Sarwar M, Hassan H, Pervez M, Fox E, 1986. Effects of fansidar on chloroquine-resistant *Plasmodium falciparum* in Pakistan. *Am J Trop Med Hyg 35:* 61–65.
218. Swellengrebel HH, 1931. Report on investigation into malaria in the Union of South Africa, 1930–31. J Med Assoc South Afr 5: 443–456.

219. Tagbor H, Cairns M, Nakwa E, Browne E, Sarkodie B, Counihan H, Meek S, Chandramohan D, 2011. The clinical impact of combining intermittent preventive treatment with home management of malaria in children aged below 5 years: cluster randomised trial. *Trop Med Int Health 16:* 280–289.
220. Tine RCK, Faye B, Ndour CT, Ndiaye JL, Ndiaye M, Bassene C, Magnussen P, Bygbjerg IC, Sylla K, Ndour JD, Gaye O, 2011. Impact of combining intermittent preventive treatment with home management of malaria in children less than 10 years in a rural area of Senegal: a cluster randomized trial. *Malar J 10:* 358.
221. Turner DA, 1977. A review of the malaria eradication programme in the Solomon Islands 1975–1976. P N G Med J 20: 188–197.
222. Usenbaev NT, Baranova AM, Annarbaev AA, Almerekov KS, 2008. Experience in sanitizing an urban focus of vivax malaria (Tashkumyr, Kyrghyzstan). *Med Parazitol (Mosk) 3:* 45–46.
223. Usenbaev NT, Yezhov MN, Zvantsov AB, Annarbaev A, Zhoroev AA, Almerekov KS, 2006. An outbreak of *Plasmodium vivax* malaria in Kyrghyzstan. *Med Parazitol (Mosk) 1:* 17–20.
224. Van Dijk WJOM, 1961. Mass treatment of malaria with chloroquine: results of a trial in Inanwatan. *Trop Geogr Med 13:* 351–356.
225. Van Dijk WJOM, 1958. Mass chemoprophylaxis with chloroquine additional to DDT indoor spraying. *Trop Geogr Med 10:* 379–384.
226. Van Goor WT, Lodens JG, 1950. Clinical malaria prophylaxis with proguanil. *Doc Neerl Indones Morbis Trop 2:* 62–81.
227. Verhoef H, West CE, Nzyuko SM, de Vogel S, van der Valk R, Wanga MA, Kuijsten A, Veenemans J, Kok FJ, 2002. Intermittent administration of iron and sulfadoxine-pyrimethamine to control anaemia in Kenyan children: a randomised controlled trial. Lancet 360: 908–914.
228. Villegas L, Cairo H, Huur A, Vinisi H, Pereira H, Jozuazoon N, Refos F, Summerville S, Hardjopawiro L, Jordaan M, Mitro S, Hiwat H, Alensi C, 2010. Mass screening and treatment for malaria among gold miners in Suriname. *Int J Infect Dis 14:* e435.
229. Von Seidlein L, Walraven G, Milligan PJM, Alexander N, Manneh F, Deen JL, Coleman R, Jawara M, Lindsay SW, Drakeley C, De Martin S, Olliaro P, Bennett S, van der Loeff MS, Okunoye K, Targett GAT, McAdam KPWJ, Doherty JF, Greenwood BM, Pinder M, 2003. The effect of mass administration of sulfadoxine-pyrimethamine combined with artesunate on malaria incidence: a double-blind, community-randomized, placebo-controlled trial in The Gambia. Trans R Soc Trop Med Hyg 97: 217–225.
230. Wallace MF, 1954. Resochin: single dose therapy and mass suppression. *Med J Malaya 8:* 251–259.
231. Wallace RB, 1934. Mass treatment with atebrin and plasmochin simplex. *Med J Malaya 9:* 33–37.
232. Watkins WM, Brandling-Bennett AD, Oloo AJ, Howells RE, Gilles HM, Koech DK, 1987. Inadequacy of chlorproguanil 20 mg per week as chemoprophylaxis for falciparum malaria in Kenya. Lancet 1: 125–128.
233. White RS, Adhikari AK, 1934. Anti-gametocyte treatment combined with anti-larval malaria control. *Rec Malar Surv Ind 4:* 77–94.
234. White RS, Adhikari AK, 1937. Anti-gametocyte treatment combined with anti-larval malaria control, part II. *Rec Malar Surv Ind 7:* 221–231.
235. Winter HG, 1934. Malaria control in Bengal. *J R Army Med Corps 63:* 238–246.
236. Wone I, Michel R, 1967. Bilan de la chimioprophylaxie systematique par chloroquine au Senegal, 1963–1966. *Med Afr Noire 14:* 249–322.
237. Yip K, 1998. Antimalarial work in China: a historical perspective. Parassitol 40: 29–38.
238. Institute for Medical Research (Malaysia), 1933. *Annual Report of the Institute for Medical Research for the Year 1932*. Kuala Lumpur, Malaysia: Govt. Press, 1933.
239. Department of Health, The Executive Yuan Republic of China, 1991. Malaria eradication in Lanyu. *Malaria Eradication in Taiwan* 245–262.

SUPPLEMENTAL APPENDIX B: STUDY EXCLUSION/INCLUSION PROCESS

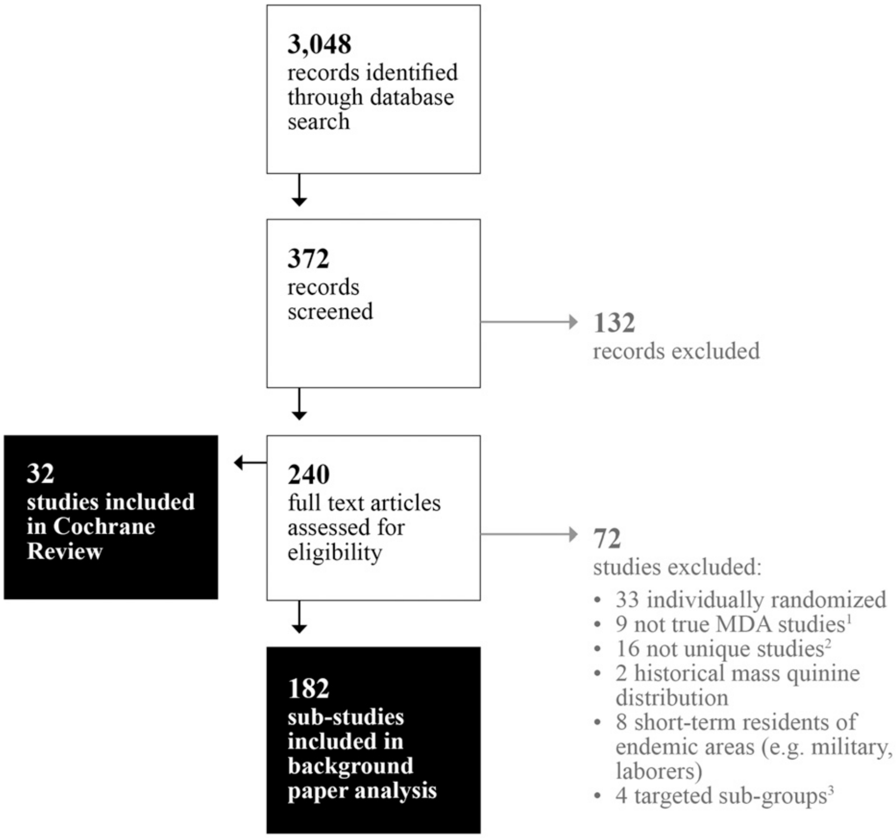

1 Studies in which the primary focus was not MDA (e.g. historical program reviews that made mention of MDA, or community surveys done in conjunction with MDA)

2 Multiple phases of the same study that were published individually

3 Treatment given at milestones rather than calendar dates (e.g. intermittent preventive treatment to infants coinciding with vaccination schedule, or to pregnant women coinciding with trimester check-ups)
